# Insertion of a Foreign Body Into the Urethra: A Report of a Rare Case

**DOI:** 10.7759/cureus.78272

**Published:** 2025-01-30

**Authors:** Georgios Haronis, Diomidis Kozyrakis, Georgios Kallinikas, Evaggelos Rodinos, Athanasios Filios, Despoina Mitiliniou, Konstantinos Safioleas, Anastasios Zarkadas, Dimitrios Bozios, Athanasios Karmogiannis, Vasileios Konstantinopoulos, Anna-Maria Konomi, Panagiotis Filios

**Affiliations:** 1 Urology, "Konstantopoulio" General Hospital, Nea Ionia, GRC

**Keywords:** endoscopy, foreign object, removal, trauma, urethra

## Abstract

Many incidents of insertion of foreign bodies into the urethra have been recorded in the literature, mainly due to self-erotic stimulation, but also have been associated with intoxication, psychiatric disorders, and senility. An accurate history is sometimes difficult to be taken due to the embarrassment and fear of the patients, delaying therefore the accurate and prompt diagnosis. Besides the patient's history and clinical examination, imaging tests like ultrasound, X-ray, magnetic resonance imaging (MRI), and computed tomography (CT), potentially accompanied by psychosexual evaluation, may guide the urologist in establishing the correct diagnosis and offering the patient the appropriate holistic treatment. In this case report, a 73-year-old man presented in the emergency department with dysuria, frequent urination, and incomplete bladder emptying. A cylindrical glass tube, which was used to test the pH of alcoholic solutions, was found broken into the posterior urethra, and endoscopic extraction was successfully performed under spinal anesthesia.

## Introduction

The insertion of a foreign body into the urethra is a rather uncommon incident without the presence of typical symptoms [1,2]. Some patients complain of dysuria, hematuria, urgency, and acute urinary retention, while, in others, more severe clinical conditions may be encountered (e.g., abscess formation), which can misguide the urologist to the diagnosis of a urinary tract infection. Inappropriate treatment may occasionally lead to urosepsis and acute kidney injury [3,4]. In extremely rare cases, the clinical course after the insertion of a foreign body into the urethra is rather asymptomatic, and the patients themselves may not be able to recall the event [1]. There are numerous cases of insertion of different kinds of foreign bodies into the lower urinary tract such as electric wires, sewing needles, pins, screws, nail clippers, mascara brushes, ballpoint pens, and olive seed [1,5-12]. Besides the patient's history and clinical examination, the urologist is guided towards the correct diagnosis by imaging tests. Most of the patients who are diagnosed with foreign objects in their urethra are males [13]. We present herein an unusual case of a 73-year-old man, who inserted a cylindrical glass tube into his urethra which was found broken therein.

## Case presentation

A 73-year-old man, from a rural area of a country of the Balkan Peninsula, presented himself to the emergency department with symptoms of dysuria, frequency, urgency, and incomplete bladder emptying. The patient originally described his lower urinary tract symptoms as long-term (lasting for several years). The communication between the patient and the doctor was difficult because he was not a native speaker; therefore, the history-taking and the description of the symptoms were provided by his companion, who served as a translator. During the physical examination, the palpation of the patient's lower abdomen, penis, and testicles was unremarkable, but a hard smooth mass of the middle line of the perineum was detected, causing moderate discomfort to the patient. This finding raised the suspicion of the presence of a perineal tumor or a foreign body in the urinary tract. The digital rectal examination revealed an enlarged, smooth, hard, and tender prostatic gland. We conducted a total blood count, which showed mild leucocytosis (16,440/μL; normal range: 4,500-10,000/μL) with an increased neutrophil level (82.3%; normal percentage: <75%), and urinalysis which revealed elevated white blood cells (>100 per high-power field (hpf); normal range: 0-2 per hpf) and red blood cells (>30 per hpf; normal range: 0-3 per hpf) but with a negative urinary culture for bacteria detection. Renal biochemistry was normal, with creatinine being 0.84 mg/dL (normal range: 0.5-1.5 mg/dL) and urea being 44 mg/dL (normal range: 20-50 mg/dL) (Table 1).

**Table 1:** Patient's blood and urine test results compared to normal values provided by our lab hpf: high-power field

Blood and urine tests	Patient's values	Lab's normal values
White blood cells	16,440/μL	4,500-10,000/μL
Neutrophils	82.3%	<75%
Creatinine	0.84 mg/dL	0.5-1.5 mg/dL
Urea	44 mg/dL	20-50 mg/dL
White blood cells in urine	>100 per hpf	0-2 per hpf
Red blood cells in urine	>30 per hpf	0-3 per hpf

**Figure 1:**
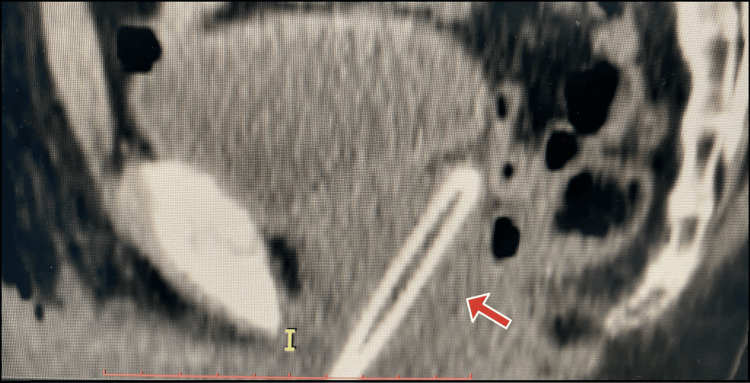
CT image showing the penetration of the prostatic gland by the inserted object CT: computed tomography

A kidney-ureter-bladder X-ray was indicative for the presence of a foreign body in the urethra, but ultrasonography of the urinary tract revealed an almost empty bladder without hydronephrosis. An abdominal computed tomography (CT) scan was subsequently performed, which established the correct diagnosis of the presence of a foreign object in the urethra sized 5 cm in length and 1.2 cm in diameter. The object was proximally perforating the prostate gland up to the capsule (Figure 1).

**Figure 2:**
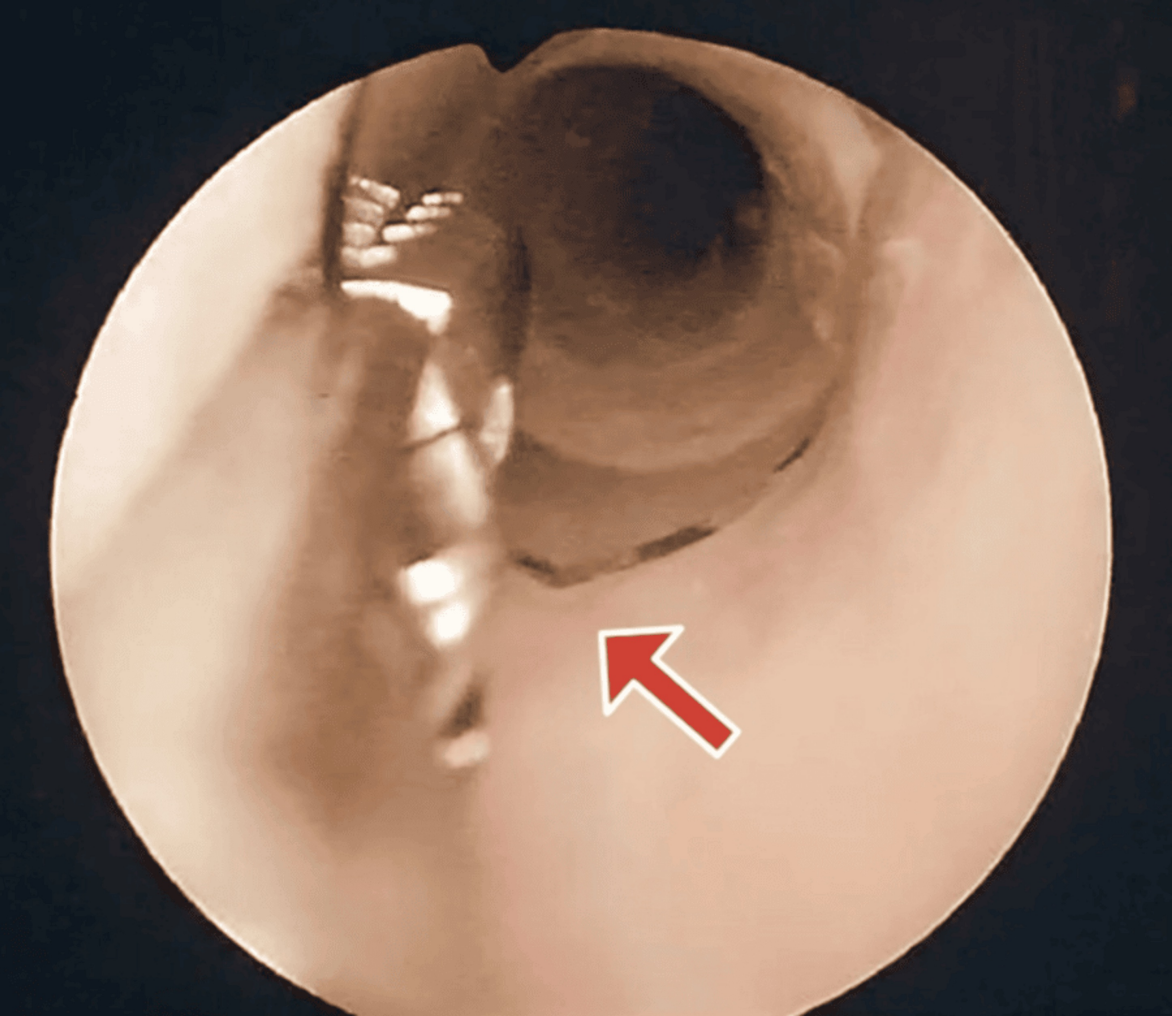
The broken distal edge of the object causing trauma to the urethra. The inserted object is rather impacted

The patient was repeatedly asked about the conditions under which the object was found in the urethra and revealed that it was himself who inserted a thermometer-like object five days ago to alleviate the symptoms of benign prostate hyperplasia. He claimed that he was instructed by a rural physician to do so in case of any exacerbation of his lower urinary tract symptoms. Afterwards, urethroscopy under local anesthesia was performed, which revealed several glass shards and debris in the urethra and, also, the main body of a broken cylindrical glass tube. Using alligator forceps, some of the shards were removed. The main foreign object seemed to be impacted (Figure 2); therefore, no further actions were undertaken under local anesthesia.

**Figure 3:**
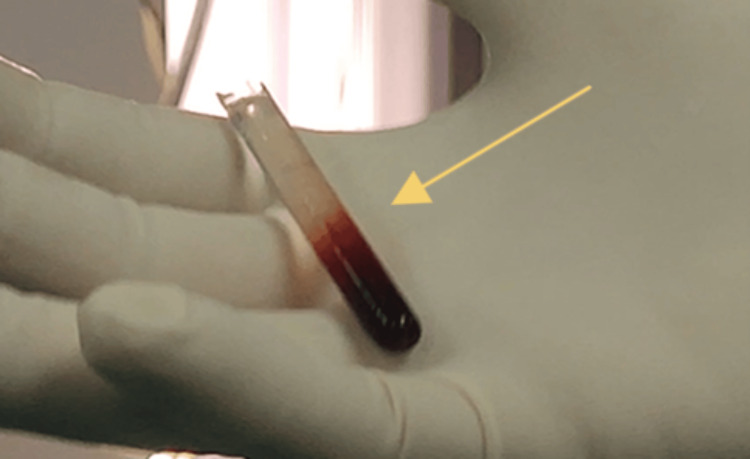
The glass-made object after its removal

**Figure 4:**
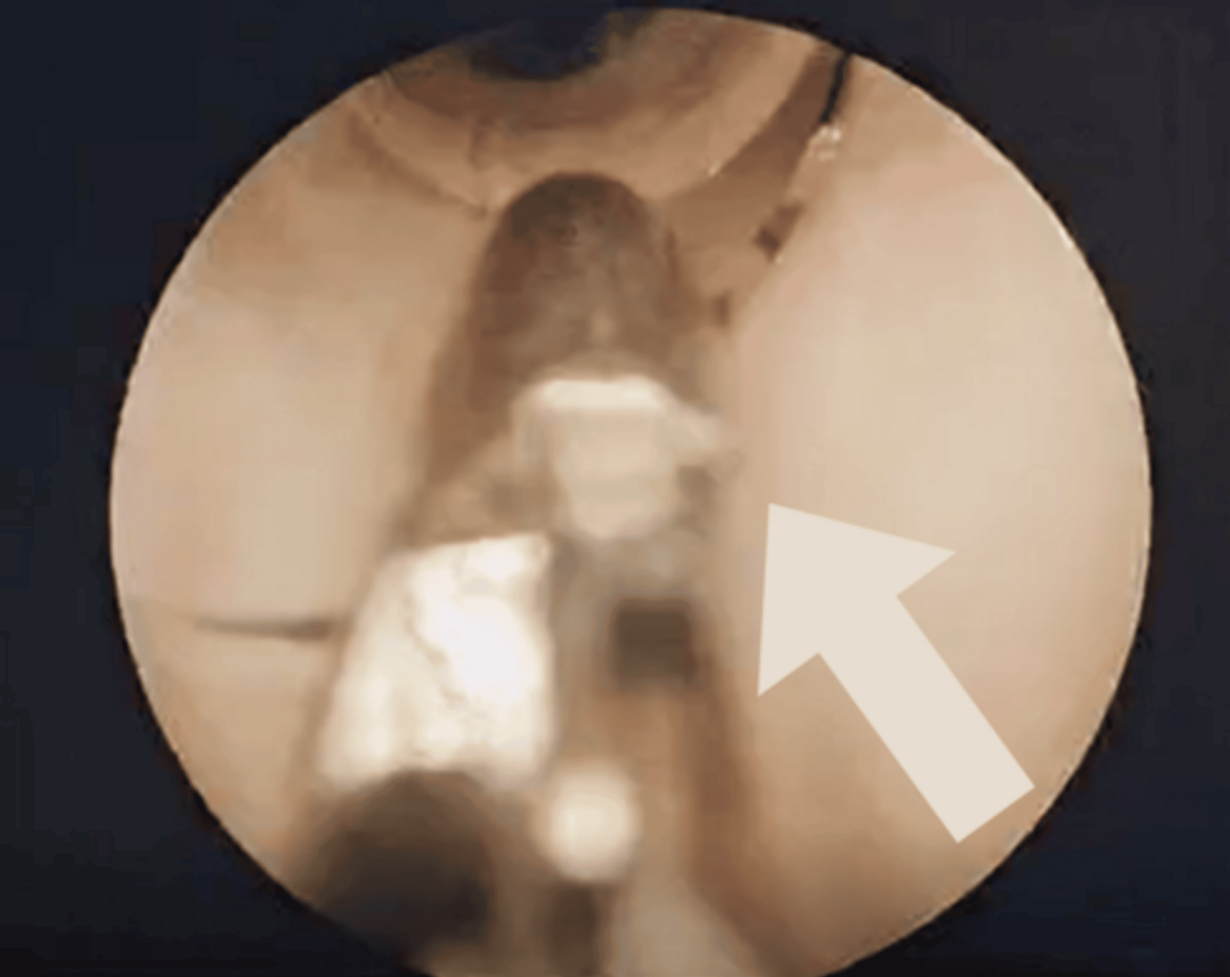
Removing the glass shards and debris through the urethra

**Figure 5:**
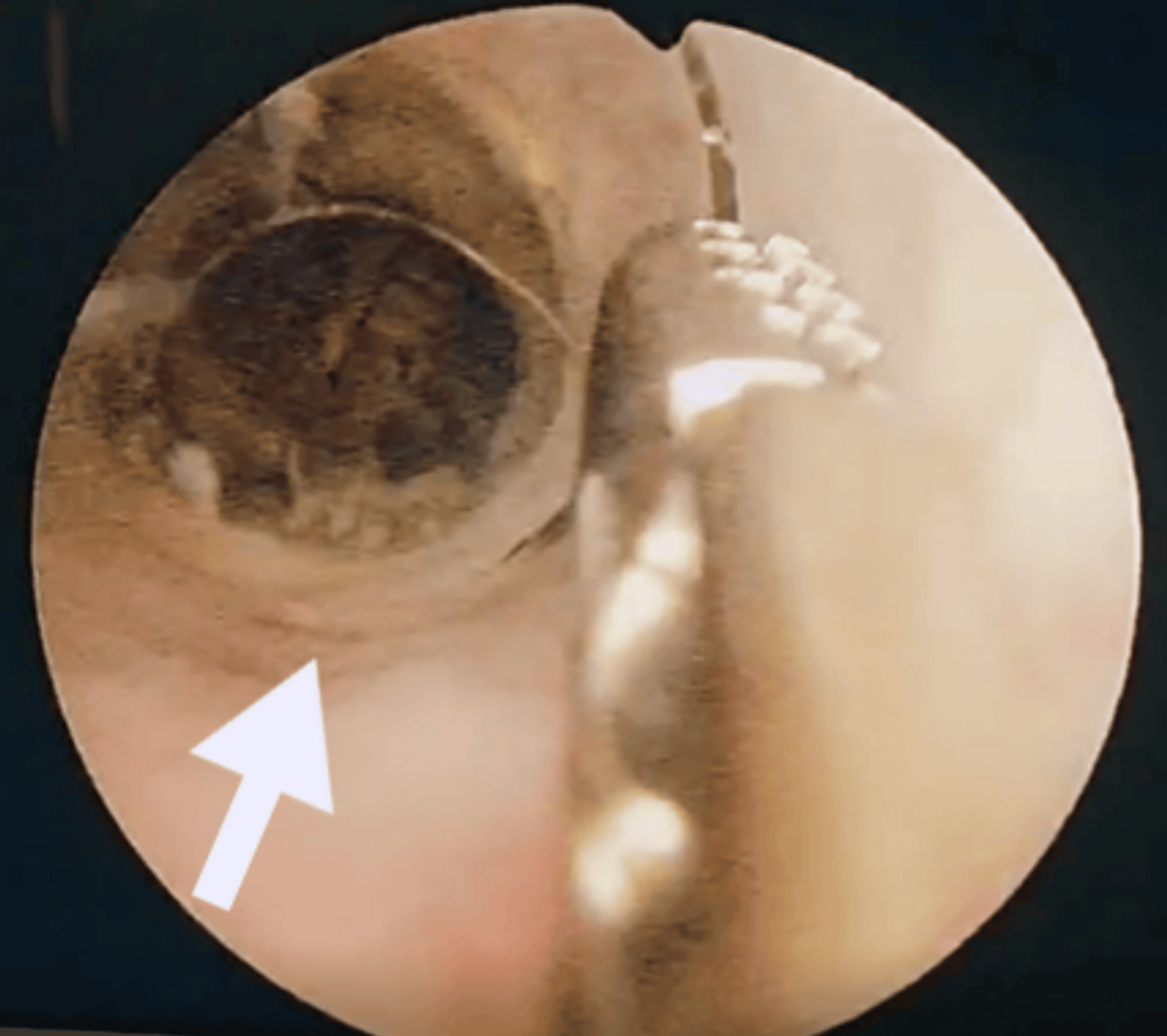
Removing the glass shards and debris through the urethra

The patient, with the translation being performed by his companion and aided by the Google Translate application, was fully informed about his health condition. He also provided an informed consent for interventional treatment under regional anesthesia. Our intention was to initially attempt the endoscopic removal of the main glass body and debris under spinal anesthesia. Should this attempt fail or be deemed of high risk for urethral trauma, an open transperineal extraction would be subsequently performed. A 26F cystoscope and alligator forceps were used to grasp and extract the object. We straightened the posterior urethra with the anterior one, by gently applying external pressure to the perineum and traction of the penis. This maneuver resulted in the disengagement of the foreign object and its extraction out of the urethra with the forceps (Figure 3). Minimum force was applied during the extraction, to avoid any further trauma to the tissues or a new breaking of the glass object. After the removal, a cystoscopy was performed to detect residual debris and remove the rest of the shards from the bladder and posterior urethra (Figure 4 and Figure 5). During the endoscopic manipulations, no bleeding from the urethra mucosa was noted. Penetration of the prostatic urethra was also revealed, without active bleeding or the presence of glass fragments. Nonbleeding trauma of the bulbar urethra with signs of local healing was also noted, attributed to the pressure of the distal cutting edge of the broken glass and its longstanding dwelling. The procedure lasted approximately one hour and was completed with the placement of an 18F three-way Tiemann-Foley catheter.

Postoperatively, the patient revealed that he used to manually insert different kinds of foreign bodies into his urethra for many years, admitting that this maneuver was offering him sexual arousal and a kind of erotic pleasure. He emphasized that he was unaware of the risks of this practice and assured the physicians that he would never repeat this kind of maneuver in the future. During his hospitalization, the patient was cooperative, and no symptoms or signs of a significant mental or psychological disorder were diagnosed. Nevertheless, an immediate assessment by a psychiatrist was proposed to him, but the patient refused to be examined. He was counseled to visit a specialist in mental health on an outpatient basis for a detailed psychologic evaluation and prevention of any future desire to insert foreign bodies into his urethra.

The catheter was removed after three weeks, and a new flexible endoscopy was performed to check for any remaining debris and urethral stenosis. There were no shards, and the urethral lumen had significantly recovered from the trauma. Nine months later, the patient's voiding was described to be satisfactory, the post-void residual urine was unremarkable in ultrasonography, but he denied a follow-up uroflowmetry and cystourethroscopy as well as a psychologic consultation.

## Discussion

Patients usually insert foreign objects into their urethra for erotic stimulation and pleasure [14-16], and it seems to have a strong correlation with mental, psychological, and personality disorders. In a multicentric review performed in 2016 by Palmer et al., it was revealed that 86% of patients had previously been diagnosed with bipolar, schizoaffective, or antisocial personality traits [17]. Moreover, in six of the patients, 14 different insertion events were recorded; four of them inserted a foreign object twice and two others three times. This finding emphasizes the potential need for the early detection and evaluation of an underlying psychological or mental disorder. These patients may expose themselves to severe urethral trauma due to the increased risk of multiple insertions [18]. Many patients often feel embarrassed or uncomfortable talking about their sexual behaviors with their physicians, leading to a delay in the correct diagnosis and treatment and also increasing the likelihood for a complex open surgical treatment [19].

Before attempting any removal, the characteristics of the object (size, shape, location, material, mobility) must be clarified. Palmer et al. presented their experience with a series of 35 cases with foreign bodies in the urethra. In 19 of them (54%), the objects were milked out from the urethra; in seven others (20%), the objects were removed during voiding; and eight patients (22%) underwent cystourethroscopy during which the objects were removed with alligator forceps or stone baskets. In only one patient (2%), an open cystotomy was necessitated, due to the insertion of a cable during masturbation, which was rather too big to be safely removed endoscopically [17]. Bogdanović et al. proposed some criteria that must be met for a manual removal to be safely attempted. These criteria are an object of small size (<1 cm) with a smooth surface, mobile, and palpable, located in the distal urethra with no urethrorrhagia. They suggest that open surgery should be reserved for those cases only where endoscopic removal either has failed or is likely to fail and potentially cause additional harm to the lower urinary tract [15]. In line with this notion are other authors too, who suggest that most of the cases could be treated endoscopically under the condition that the extraction maneuvers are performed very carefully and precisely in order to avoid additional injury to the urethral lumen [18,20]. For more complex cases, retrograde endoscopic removal may be aided by a concomitant antegrade procedure. Albakr et al. [21] managed to successfully remove a plastic foreign body by combining simultaneously retrograde urethroscopy and percutaneous cystoscopy. If endoscopy fails, the therapeutic options depend on the location; urethrotomy is proposed for foreign bodies located in the pendulous urethral and suprapubic cystotomy for objects located in the posterior urethra and bladder [17,19]. These open procedures must be performed only in selected cases, because of the increased likelihood of complications, such as hematomas, abscess formation, infection, urinary incontinence, urethral diverticulum, or stricture, fistula, and erectile dysfunction [22,23].

This article has some limitations. It is a case report only, and the results of the present case should be generalized and applied to other similar cases with caution. Further studies are required for safer conclusions to be extracted. Nevertheless, sharing similar reports might be beneficial in enhancing the scientific experience regarding the management of patients with objects inserted into the lower urinary tract.

## Conclusions

The insertion of foreign objects into the urethra is a condition that should be managed with caution. Any manipulation in the lower urinary tract by the physician should be carefully designed and balanced in terms of risks and benefits. Irrespective of the method of treatment, the applied force during the extraction should be minimal to avoid additional trauma to sensitive tissues. Imaging tests are helpful in determining the optimal therapeutic management. If it is possible, a gentle manual or endoscopic removal may be attempted leaving the open procedure to more complex cases. A holistic therapeutic approach should be offered to the patient with the physician keeping an open mind, understanding his or her choices, and avoiding any kind of criticism for his or her actions. Considering that similar behaviors are strongly associated with psychiatric disorders, it is very important to evaluate one's motivations and psychosocial issues and refer him or her to a psychiatrist if needed.
